# Supplementary material for: Hepatic Nfe2l2 Is Not an Essential Mediator of the Metabolic Phenotype Produced by Dietary Methionine Restriction
Source: Nutrients. 2021 May 24;13(6):1788. doi: 10.3390/nu13061788 (PMC8225036; doi:10.3390/nu13061788)
Supplement: Supplementary file 1 [file nutrients-13-01788-s001.zip › nutrients-1200810-figure.pdf]

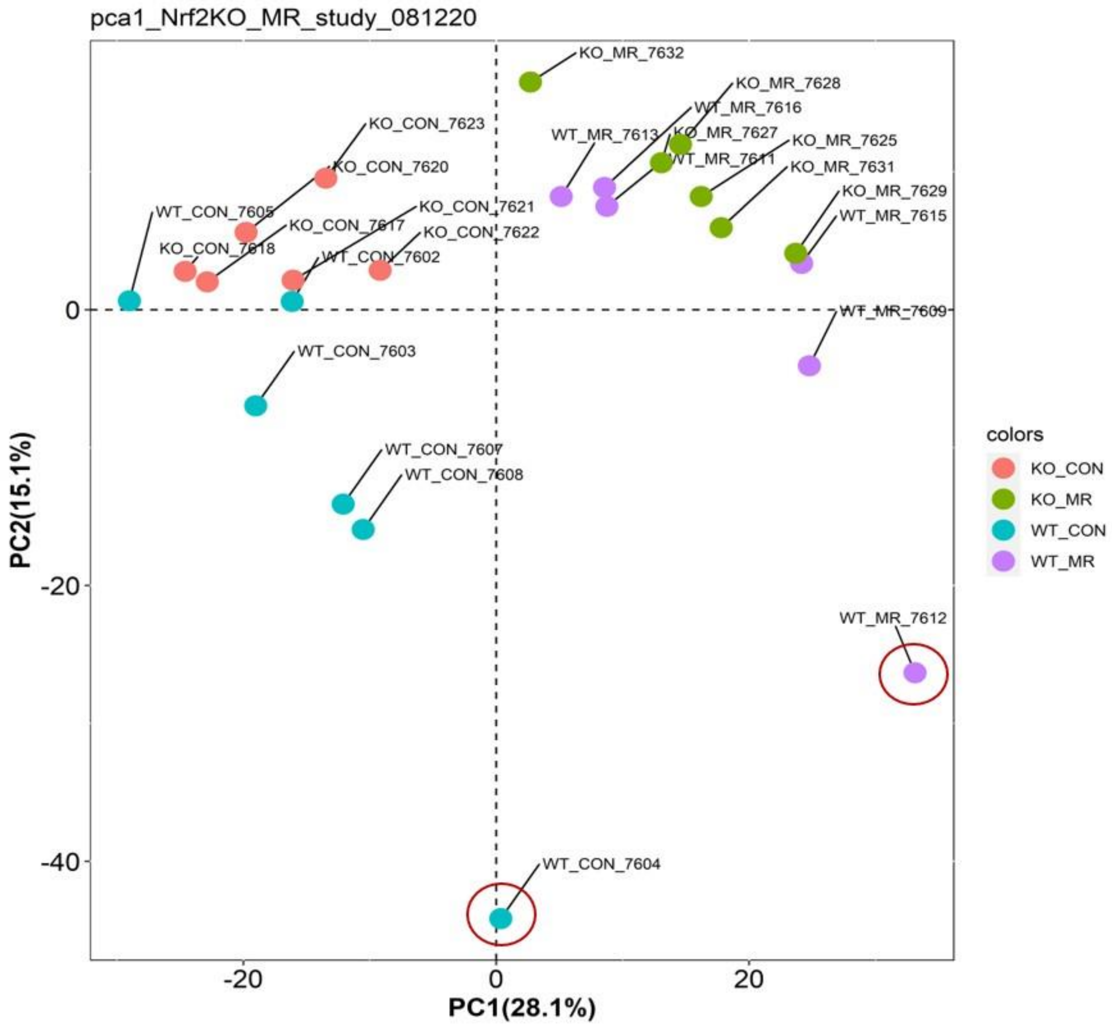

Supplementary Figure 1: Scaled normalized count data for samples from the 4 treatment groups (WT Con, WT MR, *NRF2* KO Con, and *NRF2* KO MR) were analyzed via principal component analysis (PCA) (using *prcomp* package in R, http://www.R-project.org/) to cluster samples based on gene expression similarities, and to identify potential outliers. After removal of two outlier samples (see circled WT Con and WT MR samples), differential analysis of RNA read count data was performed using DESeq2 software. Gene expression signals were logarithmically transformed (to base 2) for all downstream analyses, and genes with an absolute log fold-change ≥ 1 and false discovery rate (FDR) of 5% were considered as differentially expressed.

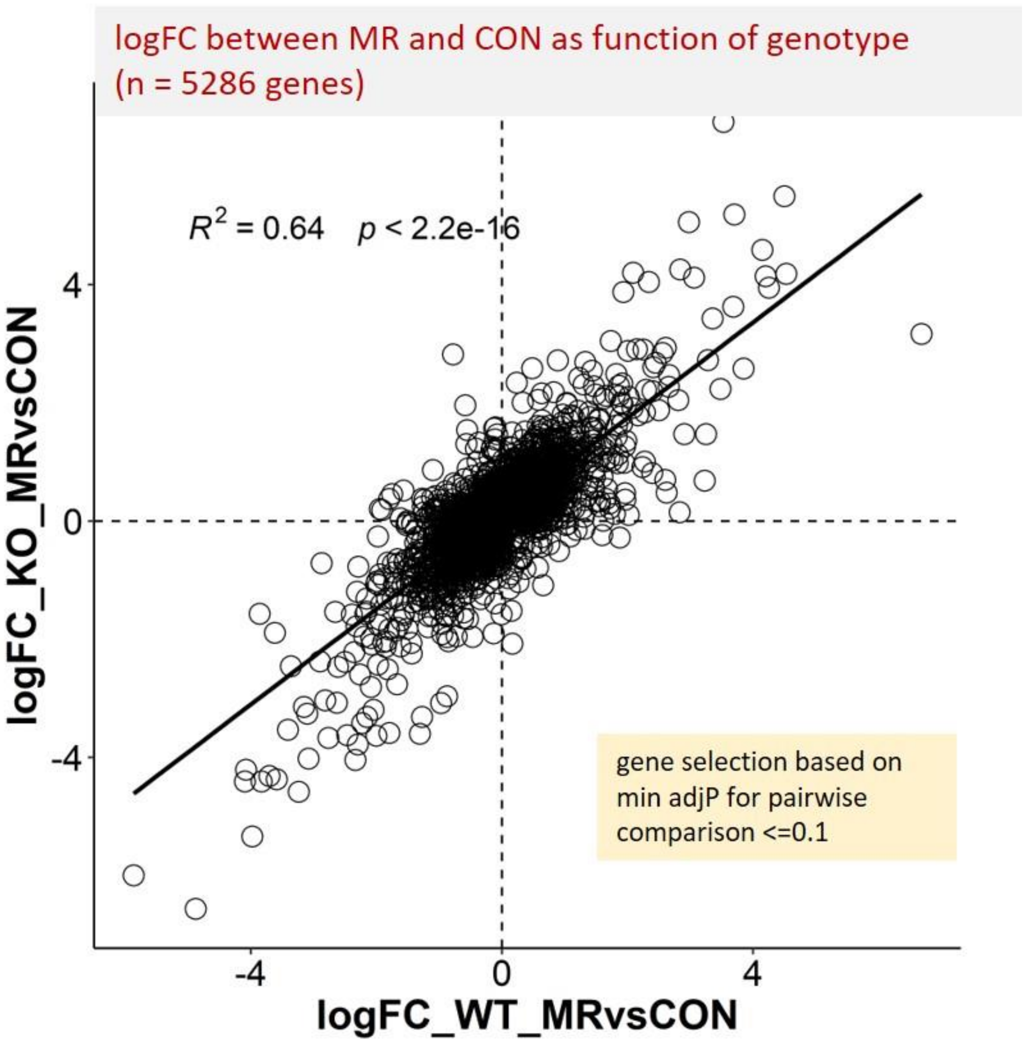

Supplementary Figure 2: Comparison of differentially expressed genes in WT MR versus WT Con and *Nrf2* KO MR versus *Nrf2* KO Con samples using a pairwise comparison of 5286 genes with adjP ≤ 0.1 for log fold change of WT MR versus WT Con and *Nrf2* KO MR versus *Nrf2* KO Con. The correlation of the effect of MR in the two genotypes was highly significant (R^2=0.64, p < 2.2 e-16).

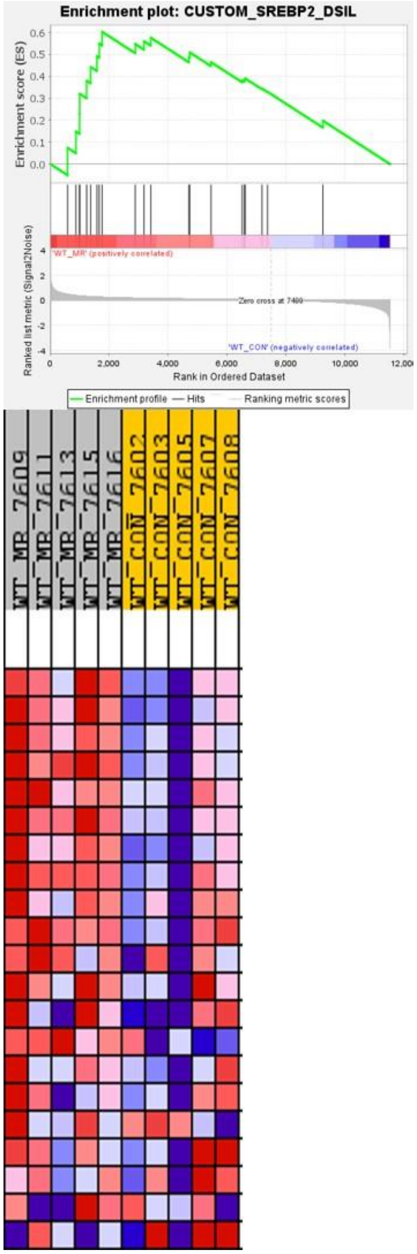

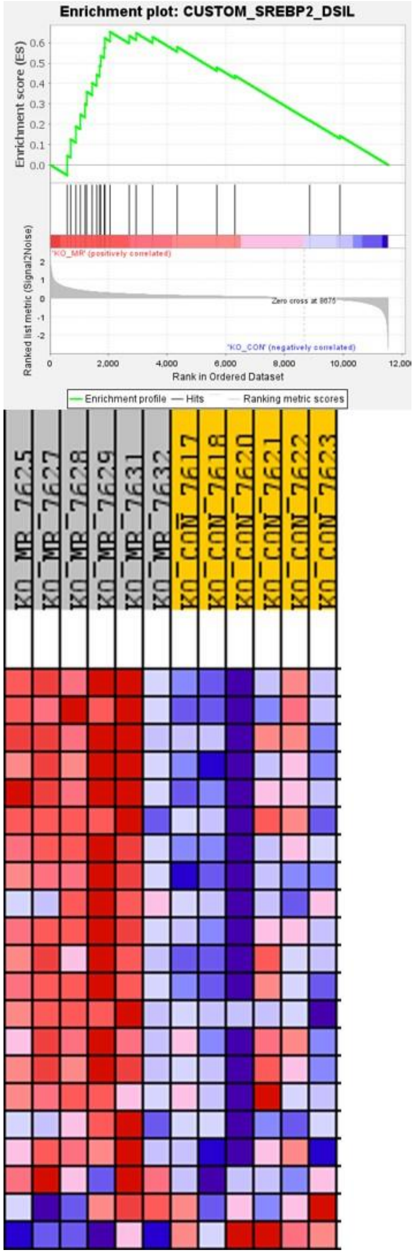

3A **3B**

Supplementary Figure 3: Gene Set Enrichment Analysis (GSEA) of hepatic gene expression in *Nfe2l2*$^{fl/fl}$ and *Nfe2l2*$^{fl/(Alb)}$ mice showing the differential effect of genotype and diet on the top scoring KEGG SREBP2 DSIL gene set. Enrichment was computed as described in the Material and Methods and the individual contributions of pathway genes to the pathway enrichment signal were visualized via enrichment plots depicting the trajectory of a normalized pathway enrichment score against the rank of the pathway genes in the context of the full gene list. Accompanying heat maps present the normalized enrichment scores for individual genes within the gene set [Blue=downregulation, Red= upregulation, Gray= not significant (FDR>0.1)]. Panel **3A** shows the enrichment set for the WT MR to WT Con comparison, and panel **3B** shows the enrichment set for the Nrf2 KO MR to Nrf2 KO Con comparison.
