# Supplementary material for: Hepatic Nfe2l2 Is Not an Essential Mediator of the Metabolic Phenotype Produced by Dietary Methionine Restriction
Source: Nutrients. 2021 May 24;13(6):1788. doi: 10.3390/nu13061788 (PMC8225036; doi:10.3390/nu13061788)
Supplement: Supplementary file 1 [file nutrients-13-01788-s001.zip › TableS1 primers.pdf]

Supplementary Table 1. Primer sequences used for qPCR

Gene	Forward	Reverse
Aox1	CTGTACCCTCAACTTACTGTCC	ACACAAACTCCCACTTCCTG
Gclc	GATGATGCCAACGAGTCTGA	GACAGCGGAATGAGGAAGTC
Gsr	CACGACCATGATTCCAGATGTT	CCGTCTGAATGCCCACTTTAT
Txnrd1	ACAGCGAGGAGACCATAGA	CCACGGTCTCTAAGCCAATAG
Trb3	CCCGGTGCCGCAGCACTTTA	GCTCGCATCTTGTCCTGGAGCC
Atf4	GGAATGGCCGGCTATGG	TCCCGGAAAAGGCATCCT
Xbp1s	CTGAGTCCGAATCAGGTGCAG	GTCCATGGGAAGATGTTCTGG
Ephx1	CGACTCGATCCAGAAACTAC	ACAGGACACATTGCAGAGAG
Sod2	CAGATTGCTGCCTGCTCTAA	CTGAAGGTAGTAAGCGTGCTC
Gsta2	GAAGGACATGAAGGAGAGAGC	TTCTTCGATTTGTTTTGCATC
Cbr1	CCGAGATGTCTGCAAGGAG	CTCACCATGCTGGACACATT
Mgst3	ATGGCTGTCCTCTCTAAGGAG	CTCTACCTTGTACTTCTTGCGG
Nqo1	AGCTGGAAGCTGCAGACCTG	CCTTTCAGAATGGCTGGCA
Asns	GGGGGCCTGGACTCGAGCTT	TTGCCACCTTTCTAGCGGCCA
Cyp4a14	CAGCTACCAAGGCAGTGTTCAG	GGACAAACGTCCATCAGAGGAC
Fgf21	TGACACCCAGGATTTGAATGAC	GCAGCCAATGATGTGTGCTTAC
Psat1	CAGTGGAGCGCCAGAATAGAA	CCTGTGCCCCTTCAAGGAG
Vldlr	TGAGCAGTGTGGCCGTCAGC	TCGGCAGGTTCGAGAAGGGCAG
Cyclophilin	CTTCGAGCTGTTTGCAGACAAAGT	AGATGCCAGGACCTGTATGCT
Nfe2l2ex5	CCATTTACGGAGACCCACCGCCTG	CTCGTGTGAGATGAGCCTCTAAGCGG
